# Top 100 most cited articles on anterior cervical discectomy and fusion

**DOI:** 10.3389/fsurg.2022.1000360

**Published:** 2022-09-06

**Authors:** Zhiyu Ding, Yijun Ren, Hongqing Cao, Jinsong Li

**Affiliations:** ^1^ Department of Orthopaedics, The Third Xiangya Hospital, Central South University, Changsha, China; ^2^ Department of Neurology, Xiangya Hospital, Central South University, Changsha, China; ^3^ Department of Spine Surgery, The Third Xiangya Hospital, Central South University, Changsha, China

**Keywords:** anterior cervical discectomy and fusion, ACDF, bibliometric analysis, most cited articles, top 100

## Abstract

**Study Design:**

Bibliometric analysis.

**Objective:**

Anterior cervical discectomy and fusion (ACDF) is a typical surgical method in spine surgery and has progressed significantly in the last several decades. The purpose of this study is to determine how the 100 most-cited original articles on ACDF have been the most influential in this field by identifying and analyzing them.

**Methods:**

The articles on ACDF were identified by searching the Thomson ISI Web of Science database on 30 May 2022. The 100 most-cited articles were selected according to specific criteria. The data extracted from the articles included title, publication date, total citations, journal name, first author, institutions, and keywords.

**Results:**

The total number of citations was 13,181, with a mean number of 131.81 ± 100.18. The publication dates ranged from 1994 to 2018. Most of these articles originated in the United States (68%) and were published in the 2000s (32%) and 2010s (48%). Spine published most of the articles (30%), followed by the Journal of Neurosurgery-Spine (16%), Spine Journal (14%), and European Spine Journal (13%). The most prolific author was Dr. Todd J Albert (*n* = 7), with 1,312 citations. The Texas Back Institute was the most productive institution (*n* = 10). The keywords ACDF, cervical spine, cervical spine, and fusion showed the highest degree of centrality.

**Conclusion:**

One hundred top-cited articles on ACDF were identified and analyzed in this study. We demonstrate that ACDF is a growing and popular area of research, with the focus of research varying through timeline trends. This will provide a comprehensive and detailed basis for spine surgeons to make clinical decisions and assimilate the research focus of cervical spine surgery.

## Introduction

Degenerative Cervical Myelopathy (DCM) is the most common cervical spinal disease (1, 2). Its treatment has evolved from conservative treatment to cervical laminoplasty, posterior cervical laminotomy and fixation, and anterior cervical discectomy and fusion (ACDF), which was first reported by Cloward, Smith, and Ronbison in 1958, opening up a new frontier in cervical spine surgery (3, 4). In the subsequent 60 years, different shapes of the iliac crest bone graft were first used for interbody fusion, and as our understanding of the biomechanics of the cervical spine improved, more types of fusion devices were invited for ACDF (3–10). Since then, ACDF has been widely used in cervical spine surgeries worldwide. In the USA, the number of patients undergoing ACDF has increased from 31 per year in 2006, to 9,937 per year in 2016 (a 31,951.6% increase), and the average age of patients is on the rise (11). ACDF has been widely accepted and gained increasing attention in recent years, resulting in a plethora of research in the cervical spine field.

Reviewing past research is an important component in advancing each specific line of research. Bibliometrics is a cross-disciplinary science of quantitative analysis of all knowledge carried out through mathematical and statistical means (12, 13). Compared with traditional reviews and meta-analyses, in bibliometric analyses, quantitative analysis and statistics are used to estimate the structure and development of a specific scientific discipline (14).

In the past decades, many bibliometric analyses have been conducted to study the most cited articles and publications on ACDF or cervical spine surgery; however, the 100 most-cited articles on ACDF from 1950 to May 2022 remain to be elucidated (15, 16). In this study, we propose to use bibliometric methods to highlight the characteristics of the 100 most-cited articles on ACDF, especially in terms of research hotspots and focus. We hope that this study highlights the potential directions for future research on ACDF and cervical spine surgery.

## Methods

### Collection and allocation of data

We searched for all relevant articles on ACDF using the Web of Science database, including the Web of Science Core Collection, MEDLINE, KCI-Korean Journal Database, Russian Science Citation Index, BIOSIS Citation Index, and SciELO Citation Index. Two researchers independently identified articles for inclusion to enhance search sensitivity. The search terms were “anterior cervical discectomy and fusion” OR “ACDF” OR “anterior cervical and discectomy and fusion” OR “anterior cervical and discectomy and fusion” OR “anterior cervical decompression and fusion” OR “anterior cervical decompression and fusions” OR “anterior cervical disc fusion” OR “anterior cervical discectomy and interbody fusion” OR “anterior cervical discectomy fusion” OR “anterior cervical discectomy with fusion” OR “cervical discectomy with fusion”.

The search was performed on 30 May 2022. We obtained 2,900 articles in total, which contained all articles published from 1980 to the present. All results were sorted from highest to lowest number of citations. We exported articles with more than 50 citations to Endnote 20 (Thomson Corporation, USA) for further analysis. Two clinical doctors performed the review based on the inclusion criteria illustrated below, and the results were exported to an Excel spreadsheet. Each of the two reviewers identiﬁed the 100 most-cited articles by screening the full text. An experienced professor compared their results carefully screening for discrepancies, and the final results were generated after a group discussion for subsequent analysis.

### Eligibility criteria

The inclusion criteria were: (1) basic science research, anatomic studies, animal research, and clinical research related to ACDF; (2) diagnosis, treatment, prognosis, or epidemiologic association with ACDF; and (3) original article, review, case report, editorial, clinical trial, and any other paper type closely connected with ACDF.

### Data extraction

All the included articles were independently reviewed by the same two authors as above. The following information was recorded for all the articles: title, first author's name, journal name, year of publication, impact factor of the journal in 2021, total number of citations of the article, average citations per year, geographic origin, institutions, and author keywords.

### Replicability and reproducibility

Replication means that people independent from the initial data extraction will simulate the search, while answering the same research question; whereas reproducibility means that the data analysis will be repeated by a person not involved in the first analysis to verify selection and quality of data. Both replicability and reproducibility were verified through the author not involved in the first search, and led to the same results, hence confirming the quality of the bibliometric analysis reported in this study.

## Results

All the 100 most-cited articles are listed in Table 1 and arranged by citation rank. The total number of citations was 13,181 (mean ± SD, 131.81 ± 100.18). Of these, 7 articles were cited more than 300 times.

**Table 1:** List of the 100 most-cited articles in anterior cervical decompression and fusion.

Rank	Article	Country	Total citations	Citations in last 5 years
1	Silber J. S, Anderson D. G, Daffner S. D, et al. Donor site morbidity after anterior iliac crest bone harvest for single-level anterior cervical discectomy and fusion. *Spine* 2003; 28(2): 134–139.	United States	660	137
2	Fountas Kostas N, Kapsalaki Eftychia Z, Nikolakakos Leonidas G, et al. Anterior cervical Discectomy and fusion associated complications. *Spine* 2007; 32(21): 2310–2317.	United States	604	234
3	Mummaneni Praveen V, Burkus J, Kenneth, Haid Regis W, et al. Clinical and radiographic analysis of cervical disc arthroplasty compared with allograft fusion: a randomized controlled clinical trial. *Journal of Neurosurgery-Spine* 2007; 6(3): 198–209.	United States	437	98
4	Murrey, Daniel, Janssen, Michael, Delamarter, Rick, et al. Results of the prospective, randomized, controlled multicenter Food and Drug Administration investigational, device exemption study of the ProDisc-C total disc replacement versus anterior discectomy and fusion for the treatment of 1-level symptomatic cervical disc disease. *Spine Journal* 2009; 9(4): 275–286.	United States	403	97
5	Heller John G, Sasso, Rick C, Papadopoulos Stephen M, et al. Comparison of BRYAN Cervical Disc Arthroplasty With Anterior Cervical Decompression and Fusion Clinical and Radiographic Results of a Randomized, Controlled, Clinical Trial. *Spine* 2009; 34(2): 101–107.	United States	381	104
6	Kaiser M. G, Haid, R. W, Subach, B. R, et al. Anterior cervical plating enhances arthrodesis after discectomy and fusion with cortical allograft. *Neurosurgery* 2002; 50(2): 229–236.	United States	326	67
7	Fraser. Jusun F, Haertl. Roger. Anterior approaches to fusion of the cervical spine: a metaanalysis of fusion rates. *Journal of Neurosurgery-Spine* 2007; 6(4): 298–303.	United States	312	104
8	Matsunaga S, Kabayama S, Yamamoto T, et al. Strain on intervertebral discs after anterior cervical decompression and fusion. *Spine* 1999; 24(7): 670–675.	Japan	264	40
9	Hacker RJ, Cauthen JC, Gilbert TJ, et al. A prospective randomized multicenter clinical evaluation of an anterior cervical fusion cage. *Spine* 2000; 25(20): 2646–2654.	United States	224	37
10	Perri Brian, Cooper Martin, Lauryssen Carl, et al. Adverse swelling associated with use of rh-BMP-2 in anterior cervical discectomy and fusion: a case study. *Spine Journal* 2007; 7(2): 235–239.	United States	193	30
11	Parker Scott L, Godil Saniya S, Shah David N, et al. Assessment of the minimum clinically important difference in pain, disability, and quality of life after anterior cervical discectomy and fusion Clinical article. *Journal of Neurosurgery-Spine* 2013; 18(2): 154–160.	United States	191	109
12	Gercek E, Arlet V, Delisle J, et al. Subsidence of stand-alone cervical cages in anterior interbody fusion: warning. *European Spine Journal* 2003; 12(5): 513–516.	Canada	189	55
13	Riley LH, Skolasky RL, Albert TJ, et al. Dysphagia after anterior cervical decompression and fusion - Prevalence and risk factors from a longitudinal cohort study. *Spine* 2005; 30(22): 2564–2569.	United States	184	58
14	Oglesby Matthew, Fineberg Steven J, Patel Alpesh A, et al. Epidemiological Trends in Cervical Spine Surgery for Degenerative Diseases Between 2002 and 2009. *Spine* 2013; 38(14): 1226–1232.	United States	169	115
15	Kim Seok Woo, Limson Marc Anthony, Kim, Soo-Bum, et al. Comparison of radiographic changes after ACDF versus Bryan disc arthroplasty in single and bi-level cases. *European Spine Journal* 2009; 18(2): 218–231.	South Korea	173	37
16	Samartzis D, Shen F H, Goldberg E J, et al. Is autograft the gold standard in achieving radiographic fusion in one-level anterior cervical discectomy and fusion with rigid anterior plate fixation? *Spine* 2005; 30(15): 1756–1761.	United States	171	46
17	Marawar Satyajit, Girardi Federico *P*, Sama Andrew A, et al. National Trends in Anterior Cervical Fusion Procedures. *Spine* 2010; 35(15): 1454–1459.	United States	161	83
18	Zigler Jack E, Delamarter Rick, Murrey Dan, et al. ProDisc-C and Anterior Cervical Discectomy and Fusion as Surgical Treatment for Single-Level Cervical Symptomatic Degenerative Disc Disease Five-Year Results of a Food and Drug Administration Study. *Spine* 2013; 38(3): 203–209.	United States	161	69
19	Buttermann, Glenn Robin. Prospective nonrandomized comparison of an allograft with bone morphogenic protein versus an iliac-crest autograft in anterior cervical discectomy and fusion. *Spine Journal* 2008; 8(3): 426–435.	United States	157	32
20	Boakye M, Mummaneni *P* V, Garrett M, et al. Anterior cervical discectomy and fusion involving a polyetheretherketone spacer and bone morphogenetic protein. *Journal of Neurosurgery-Spine* 2005; 2(5): 521–525.	United States	155	22
21	Chiles BW, Leonard MA, Choudhri HF, et al. Cervical spondylotic myelopathy: Patterns of neurological deficit and recovery after anterior cervical decompression. *Neurosurgery* 1999; 44(4): 762–769.	United States	151	43
22	Matsumoto Morio, Okada Eijiro, Ichihara Daisuke, et al. Anterior Cervical Decompression and Fusion Accelerates Adjacent Segment Degeneration Comparison With Asymptomatic Volunteers in a Ten-Year Magnetic Resonance Imaging Follow-up Study. *Spine* 2010; 35(1): 36–43.	Japan	149	52
23	Vavruch L, Hedlund R, Javid D, et al. A prospective randomized comparison between the Cloward procedure and a carbon fiber cage in the cervical spine - A clinical and radiologic study. *Spine* 2002; 27(16): 1694–1701.	Sweden	148	31
24	Song Kyung-Jin, Taghavi Cyrus E, Lee Kwang-Bok, et al. The Efficacy of Plate Construct Augmentation Versus Cage Alone in Anterior Cervical Fusion. *Spine* 2009; 34(26): 2886–2892.	South Korea	146	60
25	Samartzis Dino, Shen Francis H, Matthews Don K, et al. Comparison of allograft to autograft in multilevel anterior cervical discectomy and fusion with rigid plate fixation. *Spine Journal* 2003; 3(6): 451–459.	United States	141	42
26	Sasso Rick C, Smucker Joseph D, Hacker Robert J, et al. Clinical outcomes of BRYAN cervical disc arthroplasty: A prospective, randomized, controlled, multicenter trial with 24-month follow-up. *Journal of Spinal Disorders & Techniques* 2007; 20(7): 481–491.	United States	141	30
27	Rihn Jeffrey A, Kane Justin, Albert Todd J, et al. What Is the Incidence and Severity of Dysphagia After Anterior Cervical Surgery? *Clinical Orthopaedics and Related Research* 2011; 469(3): 658–665.	United States	135	65
28	Davis Reginald J, Nunley Pierce Dalton, Kim Kee D, et al. Two-level total disc replacement with Mobi-C cervical artificial disc versus anterior discectomy and fusion: a prospective, randomized, controlled multicenter clinical trial with 4-year follow-up results. *Journal of Neurosurgery-Spine* 2015; 22(1): 15–25.	United States	133	75
29	Niu Chi-Chien, Liao Jen-Chung, Chen Wen-Jer. Outcomes of Interbody Fusion Cages Used in 1 and 2-levels Anterior Cervical Discectomy and Fusion Titanium Cages Versus Polyetheretherketone (PEEK) Cages. *Journal of Spinal Disorders & Techniques* 2010; 23(5): 310–316.	China	133	52
30	Phillips Frank M, Geisler Fred H, Gilder Kye M, et al. Long-term Outcomes of the US FDA IDE Prospective, Randomized Controlled Clinical Trial Comparing PCM Cervical Disc Arthroplasty With Anterior Cervical Discectomy and Fusion. *Spine* 2015; 40(10): 674–683.	United States	130	89
31	Davis Reginald J, Kim, Kee D, Hisey Michael S, et al. Cervical total disc replacement with the Mobi-C cervical artificial disc compared with anterior discectomy and fusion for treatment of 2-level symptomatic degenerative disc disease: a prospective, randomized, controlled multicenter clinical trial. *Journal of Neurosurgery-Spine* 2013; 19(5): 532–545.	United States	130	61
32	Veeravagu Anand, Cole Tyler, Jiang Bowen, et al. Revision rates and complication incidence in single- and multilevel anterior cervical discectomy and fusion procedures: an administrative database study. *Spine Journal* 2014; 14(7): 1125–1131.	United States	127	84
33	Jawahar Ajay, Cavanaugh David A, Kerr Eubulus J 3rd, et al. Total disc arthroplasty does not affect the incidence of adjacent segment degeneration in cervical spine: results of 93 patients in three prospective randomized clinical trials. *Spine Journal* 2010; 10(12): 1043–1048.	United States	126	30
34	Samartzis Dino, Shen Francis H, Lyon Craig, et al. Does rigid instrumentation increase the fusion rate in one-level anterior cervical discectomy and fusion? *Spine journal* 2004; 4(6): 636–643.	United States	126	27
35	Phillips Frank M, Lee Joe Y B, Geisler Fred H, et al. A Prospective, Randomized, Controlled Clinical Investigation Comparing PCM Cervical Disc Arthroplasty With Anterior Cervical Discectomy and Fusion 2-Year Results From the US FDA IDE Clinical Trial. *Spine* 2013; 38(15): E907–E918.	United States	125	52
36	Shin Dong Ah, Yi Seong, Yoon Do Heum, et al. Artificial Disc Replacement Combined With Fusion Versus Two-Level Fusion in Cervical Two-Level Disc Disease. *Spine* 2009; 34(11): 1153–1159.	South Korea	120	34
37	Ruetten Sebastian, Komp Martin, Merk Harry. Full-endoscopic anterior decompression versus conventional anterior decompression and fusion in cervical disc herniations. *International Orthopaedics* 2009; 33(6): 1677–1682.	Germany	117	61
38	Lin Qiushui, Zhou Xuhui, Wang Xinwei, et al. A comparison of anterior cervical discectomy and corpectomy in patients with multilevel cervical spondylotic myelopathy. *European Spine Journal* 2012; 21(3): 474–481.	China	113	25
39	Delamarter Rick B, Zigler Jack. Five-Year Reoperation Rates, Cervical Total Disc Replacement Versus Fusion, Results of a Prospective Randomized Clinical Trial. *Spine* 2013; 38(9): 711–717.	United States	112	56
40	Hilibrand AS, Fye MA, Emery SE, et al. Impact of smoking on the outcome of anterior cervical arthrodesis with interbody or strut-grafting. *Journal of Bone and Joint Surgery-American Volume* 2001; 83A(5): 668–673.	United States	111	42
41	Coric Domagoj, Kim Paul K, Clemente Jonathan D, et al. Prospective randomized study of cervical arthroplasty and anterior cervical discectomy and fusion with long-term follow-up: results in 74 patients from a single site. *Journal of Neurosurgery-Spine* 2013; 18(1): 36–42.	United States	109	42
42	Tumialan Luis M, Pan Jeff, Rodts Gerald E, et al. The safety and efficacy of anterior cervical discectomy and fusion with polyetheretherketone spacer and recombinant human bone morphogenetic protein-2: a review of 200 patients. *Journal of Neurosurgery-Spine* 2008; 8(6): 529–535.	United States	108	20
43	Janssen Michael E, Zigler Jack E, Spivak Jeffrey M, et al. ProDisc-C Total Disc Replacement Versus Anterior Cervical Discectomy and Fusion for Single-Level Symptomatic Cervical Disc Disease Seven-Year Follow-up of the Prospective Randomized US Food and Drug Administration Investigational Device Exemption Study. *Journal of Bone and Joint Surgery-American Volume* 2015; 97A(21): 1738–1747.	United States	106	79
44	Wu Wen-Jian, Jiang Lei-Sheng, Liang Yu, et al. Cage subsidence does not, but cervical lordosis improvement does affect the long-term results of anterior cervical fusion with stand-alone cage for degenerative cervical disc disease: a retrospective study. *European Spine Journal* 2012; 21(7): 1374–1382.	China	104	52
45	Mummaneni Praveen V, Kaiser Michael G, Matz Paul G, et al. Cervical surgical techniques for the treatment of cervical spondylotic myelopathy. *Journal of Neurosurgery-Spine* 2009; 11(2): 130–141.	United States	103	31
46	McAfee Paul C, Cappuccino Andrew, Cunningham Bryan W, et al. Lower Incidence of Dysphagia With Cervical Arthroplasty Compared With ACDF in a Prospective Randomized Clinical Trial. *Journal of Spinal Disorders & Techniques* 2010; 23(1): 1–8.	United States	103	28
47	Zhang Xuesong, Zhang Xuelian, Chen Chao, et al. Randomized, Controlled, Multicenter, Clinical Trial Comparing BRYAN Cervical Disc Arthroplasty With Anterior Cervical Decompression and Fusion in China. *Spine* 2012; 37(6): 433–438.	China	102	29
48	Floyd T, Ohnmeiss D. A meta-analysis of autograft versus allograft in anterior cervical fusion. *European Spine Journal* 2000; 9(5): 398–403.	United States	100	15
49	Shriver Michael F, Lewis Daniel J, Kshettry Varun R, et al. Pseudoarthrosis rates in anterior cervical discectomy and fusion: a meta-analysis. *Spine Journal* 2015; 15(9): 2016–2027.	United States	96	74
50	Valencia Maldonado Carlos, Diaz-Romero Paz Ricardo, Balhen Martin Claudia. Adjacent-level degeneration after cervical disc arthroplasty versus fusion. *European Spine Journal* 2011; 20: 403–407.	Spain	95	36
51	Gao Yu, Liu Ming, Li Tao, et al. A Meta-Analysis Comparing the Results of Cervical Disc Arthroplasty with Anterior Cervical Discectomy and Fusion (ACDF) for the Treatment of Symptomatic Cervical Disc Disease. *Journal of Bone and Joint Surgery-American Volume* 2013; 95A(6): 555–561.	China	94	29
52	Jagannathan Jay, Shaffrey Christopher L, Oskouian Rod J, et al. Radiographic and clinical outcomes following single-level anterior cervical discectomy and allograft fusion without plate placement or cervical collar. *Journal of Neurosurgery-Spine* 2008; 8(5): 420–428.	United States	93	35
53	Park Daniel K, Lin Eric L, Phillips Frank M. Index and Adjacent Level Kinematics After Cervical Disc Replacement and Anterior Fusion In Vivo Quantitative Radiographic Analysis. *Spine* 2011; 36(9): 721–730.	United States	93	25
54	Dowd GC, Wirth FP. Anterior cervical discectomy: is fusion necessary? *Journal of Neurosurgery* 1999; 90(1): 8–12.	United States	92	21
55	Park Dong-Hyuk, Ramakrishnan Prem, Cho Tai-Hyoung, et al. Effect of lower two-level anterior cervical fusion on the superior adjacent level. *Journal of Neurosurgery-Spine* 2007; 7(3): 336–340.	South Korea	92	19
56	Wirth FP, Dowd GC, Sanders HF, et al. Cervical discectomy - A prospective analysis of three operative techniques. *World Neurosurgery* 2000; 53(4): 340–346.	United States	91	26
57	Elsawaf Ahmed, Mastronardi Luciano, Roperto Raffaelino, et al. Effect of cervical dynamics on adjacent segment degeneration after anterior cervical fusion with cages. *Neurosurgical Review* 2009; 32(2): 215–224.	Italy	90	28
58	Suchomel *P*, Barsa *P*, Buchvald *P*, et al. Autologous versus allogenic bone grafts in instrumented anterior cervical discectomy and fusion: a prospective study with respect to bone union pattern. *European Spine Journal* 2004; 13(6): 510–515.	Czech Republic	89	27
59	Iyer Sravisht, Kim Han Jo. Cervical radiculopathy. *Current reviews in musculoskeletal medicine* 2016; 9(3): 272–280.	United States	88	77
60	Cabraja Mario, Oezdemir Soner, Koeppen Daniel, et al. Anterior cervical discectomy and fusion: Comparison of titanium and polyetheretherketone cages. *Bmc Musculoskeletal Disorders* 2012; 13: 172.	Germany	88	44
61	Park Moon Soo, Kelly Michael *P*, Lee Dong-Ho, et al. Sagittal alignment as a predictor of clinical adjacent segment pathology requiring surgery after anterior cervical arthrodesis. *Spine Journal* 2014; 14(7): 1228–1234.	South Korea	87	64
62	Oh Min Chul, Zhang Ho Yeol, Park Jeong Yoon, et al. Two-Level Anterior Cervical Discectomy Versus One-Level Corpectomy in Cervical Spondylotic Myelopathy. *Spine* 2009; 34(7): 692–696.	South Korea	87	25
63	Pollock Raymond, Alcelik Ilhan, Bhatia Chandra, et al. Donor site morbidity following iliac crest bone harvesting for cervical fusion: a comparison between minimally invasive and open techniques. *European Spine Journal* 2008; 17(6): 845–852.	United Kingdom	86	24
64	McAfee Paul C, Reah Chris, Gilder Kye, et al. A Meta-Analysis of Comparative Outcomes Following Cervical Arthroplasty or Anterior Cervical Fusion Results From 4 Prospective Multicenter Randomized Clinical Trials and Up to 1226 Patients. *Spine* 2012; 37(11): 943–952.	United States	86	18
65	Hisey Michael S, Bae Hyun W, Davis Reginald J, et al. Prospective, Randomized Comparison of Cervical Total Disk Replacement Versus Anterior Cervical Fusion Results at 48 Months Follow-up. *Journal of Spinal Disorders & Techniques* 2015; 28(4): E237–E243.	United States	85	41
66	Park Yung, Maeda Takeshi, Cho Woojin, et al. Comparison of anterior cervical fusion after two-level discectomy or single-level corpectomy: sagittal alignment, cervical lordosis, graft collapse, and adjacent-level ossification. *Spine Journal* 2010; 10(3): 193–199.	South Korea	85	26
67	McGirt Matthew J, Godil Saniya S, Asher Anthony L, et al. Quality analysis of anterior cervical discectomy and fusion in the outpatient versus inpatient setting: analysis of 7288 patients from the NSQIP database. *Neurosurgical Focus* 2015; 39(6): E9.	United States	84	63
68	Song Kyung-Jin, Lee Kwang-Bok, Song Ji-Hoon. Efficacy of multilevel anterior cervical discectomy and fusion versus corpectomy and fusion for multilevel cervical spondylotic myelopathy: a minimum 5-year follow-up study. *European Spine Journal* 2012; 21(8): 1551–1557.	South Korea	83	36
69	Liu Yang, Hou Yang, Yang Lili, et al. Comparison of 3 Reconstructive Techniques in the Surgical Management of Multilevel Cervical Spondylotic Myelopathy. *Spine* 2012; 37(23): E1450–E1458.	China	82	35
70	Fehlings Michael G, Arvin Babak. Surgical management of cervical degenerative disease: the evidence related to indications, impact, and outcome. *Journal of Neurosurgery-Spine* 2009; 11(2): 97–100.	Canada	82	26
71	Beaurain J, Bernard *P*, Dufour T, et al. Intermediate clinical and radiological results of cervical TDR (Mobi-C (R)) with up to 2 years of follow-up. *European Spine Journal* 2009; 18(6): 841–850.	France	82	16
72	Chang Ung-Kyu, Kim Daniel H, Lee Max C, et al. Range of motion change after cervical arthroplasty with ProDisc-C and Prestige artificial discs compared with anterior cervical discectomy and fusion. *Journal of Neurosurgery-Spine* 2007; 7(1): 40–46.	South Korea	82	11
73	Adamson Tim, Godil Saniya S, Mehrlich Melissa, et al. Anterior cervical discectomy and fusion in the outpatient ambulatory surgery setting compared with the inpatient hospital setting: analysis of 1000 consecutive cases. *Journal of Neurosurgery-Spine* 2016; 24(6): 878–884.	United States	81	69
74	Vaccaro Alexander, Beutler William, Peppelman Walter, et al. Clinical Outcomes With Selectively Constrained SECURE-C Cervical Disc Arthroplasty Two-Year Results From a Prospectivei, Randomized, Controlled, Multicenter Investigational Device Exemption Study. *Spine* 2013; 38(26): 2227–2239.	United States	81	45
75	Cho Samuel K, Riew K Daniel. Adjacent Segment Disease Following Cervical Spine Surgery. *Journal of the American Academy of Orthopaedic Surgeons* 2013; 21(1): 3–11.	United States	81	31
76	Anakwenze Okechukwu A, Auerbach Joshua D, Milby Andrew H, et al. Sagittal Cervical Alignment After Cervical Disc Arthroplasty and Anterior Cervical Discectomy and Fusion Results of a Prospective, Randomized, Controlled Trial. *Spine* 2009; 34(19): 2001–2007.	United States	81	25
77	Radcliff Kris, Coric Domagoj, Albert Todd. Five-year clinical results of cervical total disc replacement compared with anterior discectomy and fusion for treatment of 2-level symptomatic degenerative disc disease: a prospective, randomized, controlled, multicenter investigational device exemption clinical trial. *Journal of Neurosurgery-Spine* 2016; 25(2): 213–224.	United States	79	61
78	Verma Kushagra, Gandhi Sapan D, Maltenfort Mitchell, et al. Rate of Adjacent Segment Disease in Cervical Disc Arthroplasty Versus Single-Level Fusion Meta-analysis of Prospective Studies. *Spine* 2013; 38(26): 2253–2257.	United States	79	38
79	Lied Bjarne, Roenning Paal Andre, Sundseth Jarle, et al. Anterior cervical discectomy with fusion in patients with cervical disc degeneration: a prospective outcome study of 258 patients (181 fused with autologous bone graft and 77 fused with a PEEK cage). *Bmc Surgery* 2010; 10: 10.	Norway	79	22
80	Liao Jen-Chung, Niu Chi-Chien, Chen Wen-Jer, et al. Polyetheretherketone (PEEK) cage filled with cancellous allograft in anterior cervical discectomy and fusion. *International Orthopaedics* 2008; 32(5): 643–648.	China	78	22
81	van Jonbergen Hans-Peter W, Spruit Maarten, Anderson Patricia G, et al. Anterior cervical interbody fusion with a titanium box cage: early radiological assessment of fusion and subsidence. *Spine Journal* 2005; 5(6): 645–649.	Netherlands	78	15
82	Moreland Douglas B, Asch Harold L, Clabeaux David E, et al. Anterior cervical discectomy and fusion with implantable titanium cage: initial impressions, patient outcomes and comparison to fusion with allograft. *Spine Journal* 2004; 4(2): 184–191.	United States	78	12
83	Zdeblick T A, Cooke M E, Kunz D N, et al. Anterior cervical discectomy and fusion using a porous hydroxyapatite bone graft substitute. *Spine* 1994; 19(20): 2348–2357.	United States	78	11
84	van Eck Carola F, Regan Conor, Donaldson William F, et al. The Revision Rate and Occurrence of Adjacent Segment Disease After Anterior Cervical Discectomy and Fusion. *Spine* 2014; 39(26): 2143–2147.	United States	77	28
85	Nassr Ahmad, Lee Joon Y, Bashir Rubin S, et al. Does Incorrect Level Needle Localization During Anterior Cervical Discectomy and Fusion Lead to Accelerated Disc Degeneration? *Spine* 2009; 34(2): 189–192.	United States	77	25
86	Kelly Michael *P*, Mok James M, Frisch Richard F, et al. Adjacent Segment Motion After Anterior Cervical Discectomy and Fusion Versus ProDisc-C Cervical Total Disk Arthroplasty. *Spine* 2011; 36(15): 1171–1179.	United States	77	20
87	Liu Yang, Qi Min, Chen Huajiang, et al. Comparative analysis of complications of different reconstructive techniques following anterior decompression for multilevel cervical spondylotic myelopathy. *European Spine Journal* 2012; 21(12): 2428–2435.	China	76	28
88	Nanda Anil, Sharma Mayur, Sonig Ashish, et al. Surgical Complications of Anterior Cervical Diskectomy and Fusion for Cervical Degenerative Disk Disease: A Single Surgeon's Experience of 1576 Patients. *World Neurosurgery* 2014; 82(6).	United States	75	43
89	Hisey Michael S, Zigler Jack E, Jackson Robert, et al. Prospective, Randomized Comparison of One-level Mobi-C Cervical Total Disc Replacement vs. Anterior Cervical Discectomy and Fusion: Results at 5-year Follow-up. *International journal of spine surgery* 2016; 10: 10–10.	United States	74	63
90	Hisey Michael S, Bae Hyun W, Davis Reginald, et al. Multi-center, prospective, randomized, controlled investigational device exemption clinical trial comparing Mobi-C Cervical Artificial Disc to anterior discectomy and fusion in the treatment of symptomatic degenerative disc disease in the cervical spine. *International journal of spine surgery* 2014; 8: 7.	United States	73	47
91	Villavicencio Alan T, Pushchak Evan, Burneikiene, Sigita, et al. The safety of instrumented outpatient anterior cervical discectomy and fusion. *Spine Journal* 2007; 7(2): 148–153.	United States	73	44
92	Radcliff Kris, Davis Reginald J, Hisey Michael S, et al. Long-term Evaluation of Cervical Disc Arthroplasty with the Mobi-C^©^ Cervical Disc: A Randomized, Prospective, Multicenter Clinical Trial with Seven-Year Follow-up. *International Journal of Spine Surgery* 2017; 11(4): 31.	United States	72	69
93	Gornet Matthew F, Burkus J Kenneth, Shaffrey Mark E, et al. Cervical disc arthroplasty with PRESTIGE LP disc versus anterior cervical discectomy and fusion: a prospective, multicenter investigational device exemption study. *Journal of Neurosurgery-Spine* 2015; 23(5): 558–573.	United States	72	51
94	Peolsson Anneli, Peolsson Michael. Predictive factors for long-term outcome of anterior cervical decompression and fusion: a multivariate data analysis. *European Spine Journal* 2008; 17(3): 406–414.	Sweden	72	30
95	Saifi Comron, Fein Arielle W, Cazzulino Alejandro, et al. Trends in resource utilization and rate of cervical disc arthroplasty and anterior cervical discectomy and fusion throughout the United States from 2006 to 2013. *Spine Journal* 2018; 18(6): 1022–1029.	United States	71	71
96	Memtsoudis Stavros G, Hughes Alexander, Ma Yan, et al. Increased In-hospital Complications After Primary Posterior versus Primary Anterior Cervical Fusion. *Clinical Orthopaedics and Related Research* 2011; 469(3): 649–657.	United States	70	30
97	Thorell W, Cooper J, Hellbusch L, et al. The long-term clinical outcome of patients undergoing anterior cervical discectomy with and without intervertebral bone graft placement. *Neurosurgery* 1998; 43(2): 268–273; discussion 273–264.	United States	70	7
98	Lovecchio Francis, Hsu Wellington K, Smith Timothy R, et al. Predictors of Thirty-Day Readmission After Anterior Cervical Fusion. *Spine* 2014; 39(2): 127–133.	United States	69	42
99	Peolsson A, Hedlund R, Vavruch L, et al. Predictive factors for the outcome of anterior cervical decompression and fusion. *European Spine Journal* 2003; 12(3): 274–280.	Sweden	68	19
100	Gruskay Jordan A, Fu, Michael, Basques Bryce A, et al. Factors Affecting Length of Stay and Complications After Elective Anterior Cervical Discectomy and Fusion A Study of 2164 Patients From The American College of Surgeons National Surgical Quality Improvement Project Database (ACS NSQIP). *Clinical Spine Surgery* 2016; 29(1): E34–E42.	United States	66	50

**Figure 1:**
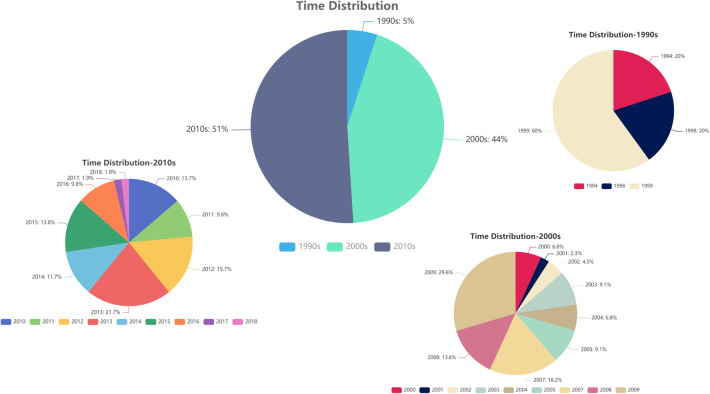
Time distribution.

The publication time ranged from 1994 to 2018, and most articles were published in the 2010s (51%) and the 2000s (44%). Meanwhile, the articles published before 2000 only accounted for 5%, and the years with the largest number of articles were 2009 (*n* = 13) and 2013 (*n* = 11) (Figure 1).

**Figure 2:**
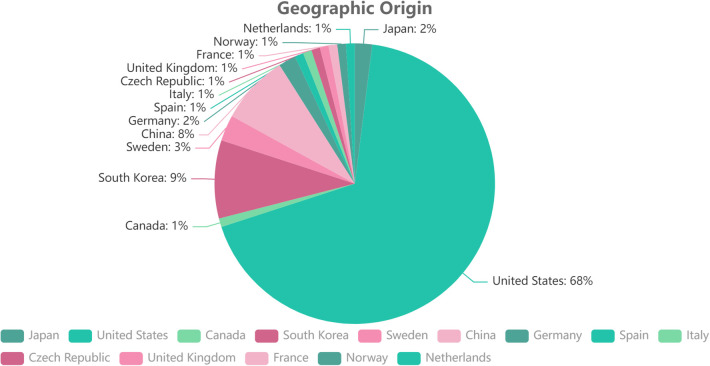
Geographic distribution.

**Figure 3:**
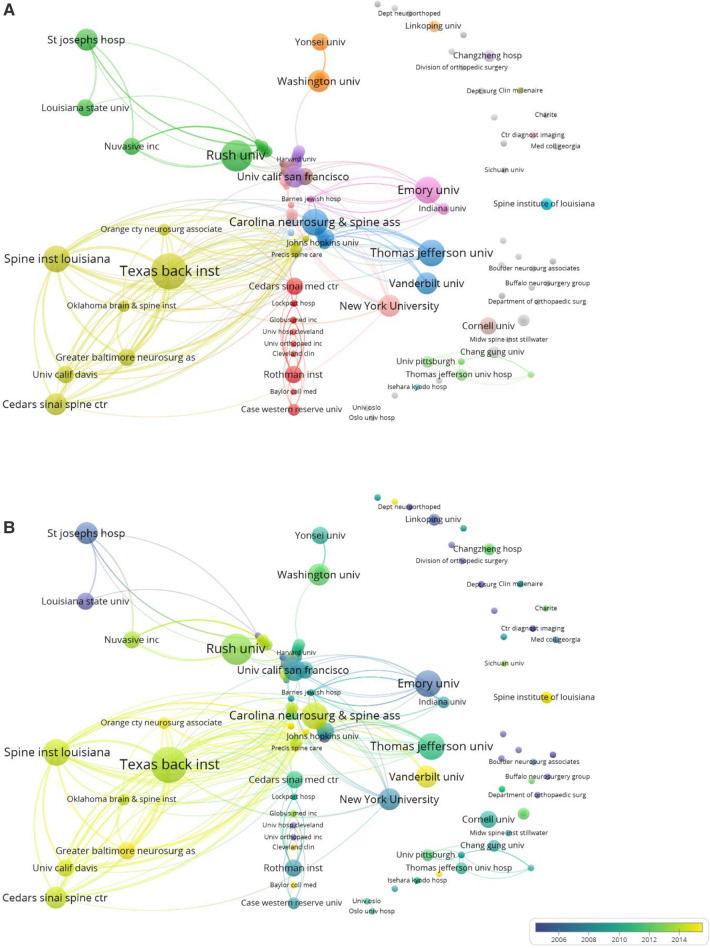
Degree of centrality analysis of the institutions of the whole 100 top-cited articles. (**A**) Overlay visualization. (**B**) Time-dependent overlay visualization.

The top 100 most-cited articles originated from 14 countries. The United States has the greatest number of published articles (*n* = 68), followed by South Korea (*n* = 9), China (*n* = 8), Sweden (*n* = 3), and Japan and Germany (*n* = 2, each). The Czech Republic, Canada, France, Italy, the Netherlands, Norway, Spain, and the United Kingdom each contributed one article (Figure 2). Regarding the institutional information, analyzed using VOSviewer, the most productive research institutions were the Texas Back Institute (TBI) and Rush University, followed by Emory University, Spine Institute of Louisiana, Carolina Neurosurg & Spine Associates, University of California San Francisco, Thomas Jefferson University, Cedars-Sinai Spine Center, and New York University. The remaining institutions are listed in Table 2 and Figure 3 according to the number of most-cited articles and published times. The cluster analysis of institutions is shown in Figure 3A, the different colors represent different clusters and the size of the spot indicates the number of institutions. Time-dependent overlay visualization of institutions is shown in Figure 3B.

**Table 2:** Institutions with multiple publications of the 100 top-cited articles on ACDF.

Institution	Country	Publications
Texas Back Institute	United States	10
Rush University	United States	9
Thomas Jefferson University	United States	8
Emory University	United States	8
Spine Institute of Louisiana	United States	7
Carolina Neurosurgery & Spine Associates	United States	7

**Figure 4:**
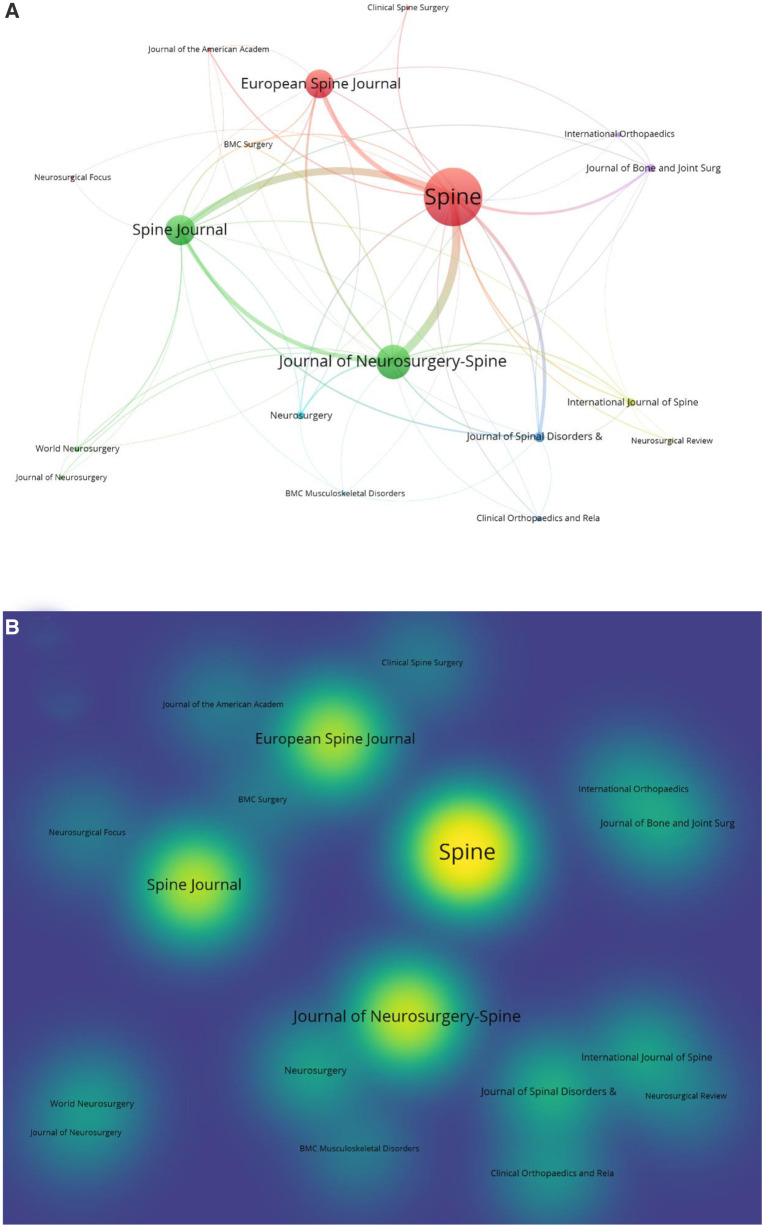
Degree of centrality analysis of the journals of the whole 100 top-cited articles. (**A**) Overlay visualization. (**B**) Hotmap overlay visualization.

All 100 top-cited articles were published in 19 journals, led by one of the most authoritative journals, Spine, which has the most publications (*n* = 30), followed by *Journal of Neurosurgery-Spine* (*n* = 16), *Spine Journal* (*n* = 14), *European Spine Journal* (*n* = 13), *Journal of Spinal Disorders & Techniques* (*n* = 4), and *Journal of Bone and Joint Surgery-American Volume*, *Neurosurgery* and *International journal of spine surgery* (*n* = 3, each); the remainder are described in Table 3. The journals and hotmap of publications were also analyzed using VOSviewer. The cluster analysis of the journals is shown in Figure 4A, where different colors indicate different clusters and the size of the spot indicates the number of articles in each journal. As for the hotmap in Figure 4B, the density of the yellow color indicates the number of articles published in every journal, showing the same result as above.

**Table 3:** Journals in which the top 100 cited articles were published.

Journal	Country	IF (2021)	No. of citations	No. of articles
*Spine*	United States	3.241	4,978	30
*Journal of Neurosurgery-Spine*	United States	3.467	2,259	16
*Spine Journal*	United States	4.297	1,841	14
*European Spine Journal*	United States	2.721	1,330	13
*Journal of Spinal Disorders & Techniques*	United States	–	462	4
*Journal of Bone and Joint Surgery-American Volume*	United States	6.558	311	3
*Neurosurgery*	United States	5.315	547	3
*International journal of spine surgery*	United States	–	219	3
*Clinical Orthopaedics and Related Research*	United States	4.755	205	2
*International Orthopaedics*	Belgium	3.479	195	2
*World Neurosurgery*	Switzerland	2.21	166	2
*Journal of Neurosurgery*	United States	5.408	92	1
*Neurosurgical Review*	Germany	2.8	90	1
*Bmc Musculoskeletal Disorders*	United Kingdom	2.562	88	1
*Current reviews in musculoskeletal medicine*	United States	–	88	1
*Neurosurgical Focus*	United States	4.332	84	1
*Journal of the American Academy of Orthopaedic Surgeons*	United States	4	81	1
*Bmc Surgery*	United Kingdom	2.03	79	1
*Clinical Spine Surgery*	United States	1.723	66	1

IF, impact factor.

Ten authors (first author, co-author, or corresponding author) published more than five publications within the top 100 most-cited articles (Table 4). The most prolific author was Todd J. Albert (*n* = 7), with a total of 1,312 citations, who is a surgeon-in-chief emeritus at the Hospital for Special Surgery and professor of orthopedic surgery at Weill Cornell Medical College (New York, NY, USA). Hyun W. Bae at the Cedars-Sinai Medical Center (Los Angeles, CA, USA) and Michael S. Hisey at the Texas Back Institute (Plano, TX, USA) were the second most prolific authors with six articles each. Alexander R. Vaccaro from the Rothman Institute, Thomas Jefferson University (Philadelphia, PA, USA) had 1,139 total citations, almost the same as Todd J Albert, who is the president of Rothman Orthopaedics and a leading doctor in spine surgery.

**Table 4:** Authors with 5 or more top-cited articles.

Author	No. of articles	Institution	Rank of articles	Total No. of citations
Todd J Albert	7	Hospital for Special Surgery and Weill Cornell Medical College, New York, NY, USA.	1, 13, 27, 46, 77, 78, 92	1,312
Hyun W Bae	6	Department of Orthopedic Surgery, Cedars-Sinai Medical Center, Los Angeles, CA, USA.	28, 31, 65, 89, 90, 92	567
Michael S Hisey	6	Department of Spine Surgery, Texas Back Institute, Plano, TX, USA.	28, 31, 65, 89, 90, 92	567
Alexander R Vaccaro	5	Department of Orthopaedic Surgery, Rothman Institute, Thomas Jefferson University, Philadelphia, PA, USA.	1, 13, 27, 74, 78	1,139
Jack E Zigler	5	Texas Back Institute and the Texas Back Institute Research Foundation, Plano, TX, USA.	4, 18, 39, 43, 89	856
K Daniel Riew	5	Department of Orthopedics, Columbia University, New York, NY, USA.	5, 61, 66, 75, 95	705
Frank M Phillips	5	Department of Orthopaedic Surgery, Rush University Medical Center, 1611 W. Harrison Street, Chicago, IL, USA.	30, 35, 46, 53, 95	522
Kee D Kim	5	Department of Neurological Surgery, UC Davis, Sacramento, CA, USA.	28, 31, 65, 89, 90	495
Reginald J Davis	5	Department of Neurosurgery, Greater Baltimore Medical Center, Baltimore, MD, USA.	28, 31, 65,90, 92	493
Greg Hoffman	5	Orthopedic North East, Fort Wayne, IN, USA.	28, 31, 65,90, 92	493

**Figure 5:**
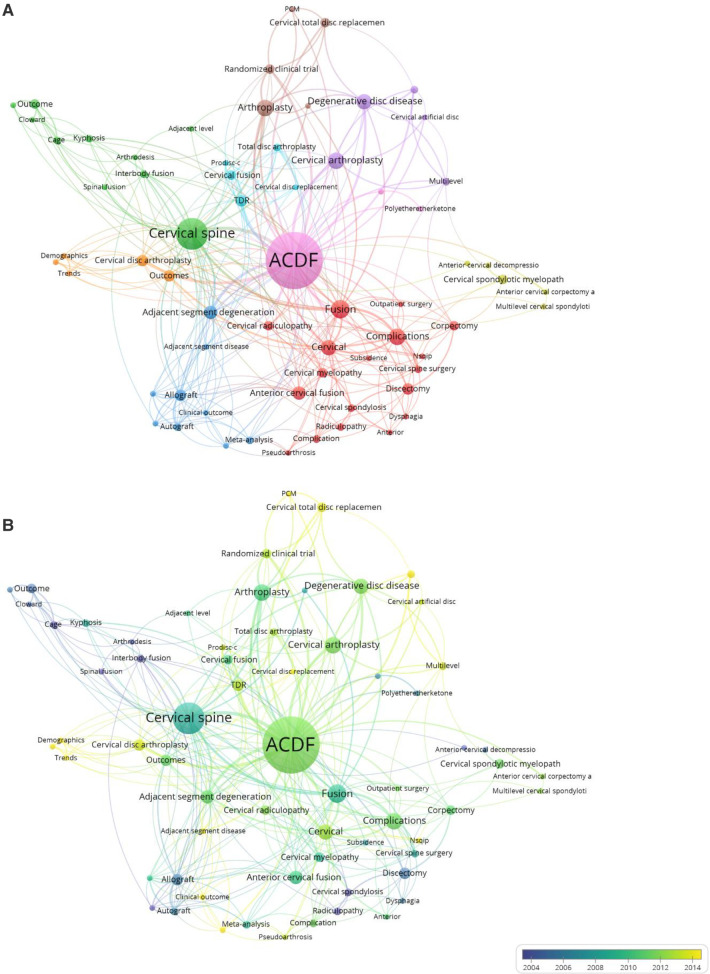
Degree of centrality analysis of the author key words of the whole 100 top-cited articles. (**A**) Overlay visualization. (**B**) Time-dependent overlay visualization.

Author keywords of all 100 articles were analyzed *via* VOSviewer network analysis, as shown in Figure 5. The results showed that except for *ACDF*, *cervical*, *cervical spine* and *fusion*, the keywords *adjacent segment degeneration*, *complications*, *cervical arthroplasty*, *degenerative disc disease*, *arthroplasty*, and *total disc replacement (TDR)* also had a higher degree of centrality (Figure 5A). Furthermore, each node is colored based on when they occurred, in a blue-to-yellow gradient (Figure 5B); this shows that except for *ACDF*, *cervical* and *complications*, the keywords *adjacent segment degeneration*, *adjacent segment disease*, *TDR*, *cervical disc replacement*, *cervical disc arthroplasty*, *cervical arthroplasty*, *prodisc-c*, *multilevel*, and *clinical outcome* have occurred in recent years.

## Discussion

The citation number is an important bibliometric indicator and a useful tool to measure the influence of publications. Many bibliometric analysis methods have also been used in various kinds of articles (17). In this study, we aimed to provide a better understanding of the historical knowledge of surgeons regarding ACDF. We also wanted to determine which articles regarding ACDF have been more impactful by identifying and analyzing the characteristics of the 100 most-cited articles.

The top 100 articles on ACDF were cited a mean of 131.81 ± 100.18 times (range, 66–660), which is more than the number of citations in other fields of spine surgery, such as endoscopic spine surgery research (mean, 84.4) (18), spinal disc arthroplasty research (mean, 115.1) (19), adult spinal deformity (mean, 34.8) (20), and idiopathic scoliosis (mean, 137.5) (21). This indicates that ACDF has been studied more frequently than other topics within the field of spine surgery.

Most of the 100 top-cited articles were published in the 2010s (51%) and the 2000s (44%), whereas only 5% were published before 2000. In our experience, older articles tend to have more citations; yet, we obtained the opposite results. This may be explained by the “obliteration by incorporation” concept, whereby a new article that originates from an early influential article gains greater popularity, reducing the citations of the original article (22). However, some researchers believe that articles may show their value 20 years after their publication (23). Moreover, some medical techniques and concepts from articles published before 2000 have been inevitably innovated by new technologies, discoveries, and views. This may explain how the 100 top-cited articles were distributed in different periods, and why the most recent article included in our list was published in 2018. Recently published research requires more time to accumulate citations and establish its significance.

As for the citations in the last five years, we found that some of the 20 top-cited articles only have a few dozen citations, such as ranks 9, 10, 15, 19, and 20 in Table 1. For several articles among the 80–100 top-cited ones, we found that the citation number for the last 5 years almost coincided with the total citation number, such as ranks 89, 90, 92, 93, 95, 98, and 100 (Table 1). Progress in academic concepts, surgical skills, and scientific research may explain this phenomenon and indicate the change in research hotspots in cervical spine surgery.

The most cited article was an investigation of donor site morbidity after anterior iliac crest bone harvest for single-level ACDF surgery by Silber Jeff S. et al. with 660 total citations after publication in 2003, which first focused on complications in the iliac crest bone graft site after single-level ACDF surgery (24). The authors found that a large percentage of patients suffered from chronic donor site pain after surgery, and long-term functional impairment could also be a significant problem. Although the study was published 20 years before, this pivotal study made surgeons aware of the need for alternative sources of graft material. Therefore, different types of interbody implants have been developed, such as hydroxyapatite (HA), polyetheretherketone (PEEK) cage, and titanium (Ti) cage, which have better shape, biomechanical function, and fusion rate (10, 25). This study greatly promoted the development of cervical interbody implants.

In 2007, Fountas Kostas et al. (26) published the second most cited article, a retrospective review, with 604 citations. This article was also about ACDF complications but focused on the ACDF surgery itself. The authors evaluated 1,015 patients undergoing first-time ACDF for cervical radiculopathy and/or myelopathy owing to degenerative disc disease and/or cervical spondylosis and analyzed the most common complications related to ACDF. This article had significant guiding significance for clinical spine surgeons in avoiding iatrogenic injury in ACDF surgery. It is worth mentioning that this article also had the most citations in the last 5 years.

The third most cited article was by Mummaneni Praveen et al. (27), which was also published in 2007. This prospective randomized multicenter study aimed to compare the clinical and radiographic outcomes between ACDF and cervical disc arthroplasty for the treatment of single-level cervical degenerative disc disease. The authors concluded that the PRESTIGE ST Cervical Disc System had more advantages in improving neurological success and clinical outcomes, as well as reducing the rate of secondary surgeries, compared with ACDF at 24 months of follow-up.

Further analysis of the articles revealed that three of the five most-cited articles were related to artificial cervical disc replacement (ADR). This kind of surgery was first proposed by Vincent E Bryan Jr in 2002 (28); since then, different kinds of movable artificial cervical discs like ProDisc-C and Mobi-C were designed and applied in clinical settings (29, 30). The Bryan disc was designed for maintaining the normal biomechanics of the cervical spine, to reduce the incidence of adjacent segment disease (ASD) and degeneration (31–33). With further research, surgeons found that the incidence of ASD was not significantly different between ACDF and ADR surgery (34, 35), but ADR surgery had the advantages of greater cervical spine mobility and less dysphagia. Moreover, the hot keywords in the 2010s were mostly about ADR surgery, which indicates that ADR surgery has huge potential in the future.

Most of the analyzed articles (68%) and journals (68.4%) originated in the United States, same as in the fields of arthroscopy (12), back pain research (36), and hand surgery (37). The inventors of ACDF, G.W. Smith, and R.B. Cloward are both from the United States, where ACDF surgery has spread worldwide. In addition, it is not unthinkable that authors from the United States are more likely to publish in US journals and usually prefer to cite US articles (38).

Regarding the journals, Spine was the most popular journal in the 100 most-cited articles, with 30 articles published in it and with 4,978 citations; three of these articles had over 300 citations. Spine is one of the most well-known and relatively older journals in the field of spine surgery, which may explain why it attracts important articles and receives more citations. The latest impact factor of Spine is 3.468, with a Q1 category quartile in the orthopedic JCR category in 2020.

The Texas Back Institute (TBI) was the most productive research institution, publishing 10 of the 100 top-cited articles. TBI was established in 1977, and surgeons have made great progress in the treatment of spinal diseases in the past 45 years. Michael S. Hisey and Jack E. Zigler, both from TBI, authored all 10 articles, making them the researchers with the most publications on the top 100 list. With their team, they have made significant achievements in cervical total disc replacement (Mobi-C from *Zimmer Biomet®* and ProDisc-C from *Centinel Spine®*) and conducted long-term follow-up studies on ACDF under many aspects (33, 39–47). Frank M. Phillips from Rush University is another ACDF pioneer, who also has advanced cervical disc replacement, and these works have made his institution productive (48–52). In addition, Thomas Jefferson University is also a productive research institution in the field of ACDF, and two of its doctors, Todd J. Albert and Alexander R. Vaccaro, are the greatest scholars in spine surgery, and their contribution to the field of ACDF relates to the most common ACDF and cervical disc replacement complications in the 100 top-cited articles (24, 34, 47, 50, 53–56). The scholars mentioned above provided great help and guidance for spine surgeons performing ACDF.

As expected, the most common keywords were *ACDF*, *fusion*, and *cervical spine*. Apart from these, *adjacent segment degeneration*, *complications*, *cervical arthroplasty*, *degenerative disc disease*, *arthroplasty*, and *TDR* were also frequently used keywords in the 100 top-cited articles. In chronological terms, the keywords *adjacent segment degeneration*, *adjacent segment disease*, *TDR*, *cervical disc replacement*, *cervical disc arthroplasty*, *cervical arthroplasty*, *prodisc-c*, *multilevel*, and *clinical outcome* were the most frequently used after 2010.

Surgery complications were always an important topic (57), and it was truly a trending research topic keyword before 2010; however, these keywords were featured relatively less often in the top 100 most-cited articles after 2010. This may be because most complications have been avoided with the directions from previous research and the development of surgical skills. The same phenomenon was observed for keywords such as *cage*, *interbody fusion*, *cervical fusion*, *allograft*, and *anterior cervical decompression*, likely for the same reason. In contrast, *cervical disc replacement*, *PCM*, *Prodisc-c*, *TDR*, *adjacent segment disease*, and *multilevel* became hot keywords after 2010. To our knowledge, adjacent segment degeneration after ACDF mostly depends on cervical biomechanical changes around the fusion level, and it could not be solved because of the principles of ACDF surgery (58–60), while ADR surgery can solve this problem in a targeted manner; therefore, we believe it was the reason for the change in hot keywords. The changes in other hot keywords' citation frequency, such as *multilevel* and *clinical outcomes*, may depend on the advances in diagnosis and treatment of the disease. In general, cervical disc replacement, adjacent segment disease, and clinical outcomes may be research hotspots for decades to come.

In our study, synthesis of the keywords in the top 100 most-cited articles on ACDF and all of author key words in the papers published over the last 5 years, we forecast the possible study trends in the future may include (1) new cervical interbody implants are the main objects of research, like Zero-profile intervertebral fusion system and 3D-print intervertebral fusion implants, etc.; (2) long time and large sample size follow-up research on new cervical interbody implants are needed in the future and (3) adjacent segment disease (degeneration) continues to be of interest for researchers.

This study has several limitations. First, all articles were identiﬁed according to the number of citations; therefore some new, just as relevant publications in the ﬁeld did not have the same opportunity to be cited often enough to be included in this study. Second, we did not exclude self-citation, as authors prefer to cite articles from the journal with which they intend to publish (61), and the citation number may not completely reﬂect the research quality. Third, the geographical clustering is limited by the growing level of international collaboration. Besides, the highest quality articles are more likely to compare two techniques or provide evidence-based guidance rather than just focusing on ACDF alone (62). Then, this bibliometric analysis only included published journal articles and other materials, such as clinical guidelines, meeting notes, and textbooks. Finally, authors prefer to cite articles that already have many citations while ignoring quality or content (63).

## Conclusion

This study identified and bibliometrically analyzed the 100 most-cited articles on ACDF between 1950 and 2022, including article title, authors, institutions, country, year of publication, journals, keywords, and total number of citations. Our study illustrates that ACDF is an improving and popular research field. Different types of cervical disc replacement skills, how to reduce the incidence of adjacent segment disease, and clinical outcomes may soon become research hotspots. This article provides insight into worldwide research trends and potential directions for future research on ACDF.

## Data Availability

The original contributions presented in the study are included in the article/Supplementary Material, further inquiries can be directed to the corresponding author/s.
